# “Jikoshu”: Japanese studies in the 1960s and 1970s, and international trends today

**DOI:** 10.1002/pcn5.112

**Published:** 2023-06-13

**Authors:** Toshiyuki Kobayashi

**Affiliations:** ^1^ Department of Psychiatry Jichi Medical University Tochigi Japan

**Keywords:** delusional disorder, obsessive‐compulsive disorder, odor, olfactory reference disorder, phobia

## Abstract

In the 1960s and 1970s, there was widespread discussion in Japan about the pathological experience of “unpleasant odors emanating from one's body.” This symptom is called “Jikoshu,” and this term was used in combination with various words, such as “Genkaku” (hallucination) and “Moso” (delusion), reflecting its symptomatological ambiguity. The best‐known term in the English‐language literature is *Jikoshu‐Kyofu* (*Jikoshu* phobia). By further abstracting this symptom and viewing it as a delusion‐like experience of “something leaking out of me,” egorrhea syndrome (Fujinawa) was proposed, which was considered to be partly a pathology of schizophrenia. Similar cases were characteristically observed during adolescence, and a study emerged suggesting that the syndrome was “adolescent paranoia” (Murakami), distinct from schizophrenia. However, the terms “Jikoshu‐Taiken” (*Jikoshu* experience; Kasahara et al.) and “Jikoshu‐Sho” (*Jikoshu* syndrome; Miyamoto) were proposed to emphasize the nosological ambiguity. Considered a culture‐bound syndrome unique to Japan or East Asia, *Jikoshu* received little attention in the English‐language literature apart from a 1971 study of olfactory reference syndrome (Pryse‐Phillips), which presents with similar symptoms. In recent years, research has placed this disorder within the obsessive‐compulsive spectrum, and it has been adopted as an ICD‐11 disorder under the term “olfactory reference disorder.”

## INTRODUCTION

In the 1960s and 1970s in Japan, the pathological experience of “unpleasant odors emanating from one's body” was widely discussed. Referred to as *Jikoshu* (literally, “self‐odor”), this has been called *Jikoshu‐Kyofu* (self‐odor phobia), *Jikoshu‐Genkaku* (self‐odor hallucination), and *Jikoshu‐Moso* (self‐odor delusion) depending on the symptomatological level. The terms *Jikoshu‐Taiken* ^1^ (self‐odor experience) and *Jikoshu‐Sho* ^2^ (self‐odor illness or self‐odor syndrome) were also proposed, highlighting the nosological and symptomatological ambiguity.

In a typical *Jikoshu* case, the patient begins to think, “People around me somehow seem to be avoiding me. When I approach them, they bring their hands to their noses, frown, or move away from me. There must be some kind of a bad smell coming out of my body.” The symptoms persist for a long time. The patient might become extremely worried and seek self‐care to eliminate the odor or seek treatment for an illness that they believe is the cause of the odor.^2^

The psychopathological study of *Jikoshu* began in 1961 with Hiroshi Adachi's case study^3^ regarding the symptom “I am emitting disgusting odors,” and was followed by many reports from various viewpoints during the 1960s and 1970s. These subsequent discussions reflected several views, including that the symptom is a phobia or obsession with one's own body odor, that the symptom represents a delusion about body odor with the overall picture seen as an adolescent psychosis distinct from schizophrenia, and that the condition is a unique clinical entity related to smell and cannot be dismissed as a hallucination or delusion.

This paper is one of a series of reviews introducing Japanese psychopathology research to the English‐speaking world.^4^, ^5^, ^6^ The aim here is to introduce *Jikoshu* studies from the 1960s and 1970s, touching on concepts such as egorrhea syndrome, adolescent paranoia, and *Jikoshu‐Sho* while also referring to recent international trends.

## *JIKOSHU* AS A PHOBIA

If the symptom “I am emitting an unpleasant odor” is described without diagnostic prejudice, it would be called *Jikoshu‐Taiken* (self‐odor experience). However, this experience was initially considered a symptom of *Taijin‐Kyofu* (or *Taijin‐Kyofu‐Sho*). Although similar to social anxiety disorder, *Taijin‐Kyofu* is a clinical entity unique to Japan and already discussed to some extent in English‐language literature. According to Takahashi,^7^ the term *Taijin‐Kyofu* first appeared in Morita's 1932 article “Erythrophobia (or *Taijin‐Kyofu*) and its treatment.”^8^ Morita described “fear of shame” as a derivative of erythrophobia: “Recently, someone had named this fear of shame *Taijin‐Kyofu*.” Morita's fear of shame or *Taijin‐Kyofu* described cases of anguish and pessimism about one's cowardice and shyness. In 1938, Takara^9^ included in *Taijin‐Kyofu* those cases involving relational ideation, such as “fear of self‐facial expression,” in which one's odd and unpleasant facial expressions is perceived to cause discomfort in others and others are experienced as having unpleasant facial expressions. From the 1950s onward, a series of articles was published on the relationship between *Taijin‐Kyofu* and Japanese social culture, which spread understanding of *Taijin‐Kyofu* as a uniquely Japanese neurosis.

Subsequently, similarities between *Taijin‐Kyofu* and DSM (*Diagnostic and Statistical Manual of Mental Disorders*)^10^, ^11^ social anxiety disorder were recognized, and so recent descriptions of *Taijin‐Kyofu* were likely written with DSM social anxiety disorder in mind. However, as the concept of social anxiety disorder was introduced to Japan, it was realized that there were two subtypes of *Taijin‐Kyofu*.^12^ One subtype is generally equivalent to the DSM concept,^10^, ^11^ which Nagata et al.^13^ termed the typical subtype. The other is what they called the offensive subtype, which is more in line with the older description in *Taijin‐Kyofu* (Table 1 briefly summarizes the characteristics of the offensive subtype according to Yamashita^12^). For example, Shinfuku's 1958 textbook^14^ states the following: “In our country, there are often patients who have a pathological fear and avoidance of going out in public, being in public, and interacting with others, collectively referred to as *Taijin‐Kyofu*. The basis of this symptom varies from case to case, but can be broadly classified into the following categories: fear of looking at others (*Jiko‐Shisen‐Kyofu*), fear of one's own face (blushing, etc.) and attitude (erythrophobia, fear of getting nervous), and fear of unpleasant body odor (*Jikoshu‐Kyofu*).” Whereas social anxiety disorder is a problem of anxiety in social situations, Shinfuku's description suggests that the core of *Taijin‐Kyofu* is a phobia of various phenomena that occur in interpersonal situations, with *Jikoshu‐Kyofu* occupying a significant part of the picture.

**Table 1:** Characteristics of offensive‐type of *Taijin‐Kyofu* (Yamashita).^12^

1. Firm belief in the existence of serious shortcomings (such as flushing, bodily odors). 2. Its existence is intuitively perceived by patients from the behavior, actions, and gestures of other people around them. 3. These defects make others to feel unpleasant and therefore must be corrected or removed. 4. No other symptoms appear in the long follow‐up period of time.

This type of fear, also referred to as the offensive‐type of *Taijin‐Kyofu*, is the fear that certain features of the patient might be objectionable or harmful to society. As mentioned above, Japanese textbooks from the mid‐20th century indicate the offensive‐type was considered the essence of *Taijin‐Kyofu*. In fact, the offensive‐type was considered a culture‐bound syndrome characteristic of East Asia. Although some studies have suggested the specific involvement of cultural characteristics,^15^ others considered the offensive‐type not so different from social anxiety disorder.^16^, ^17^ In one study, avoidance of eye contact was associated with social anxiety disorder,^18^ whereas in another study, *Jiko‐shisen‐Kyofu* not *Taijin‐Kyofu* was considered specific to East Asia.^19^ There might be a growing recognition that *Taijin‐Kyofu* and social anxiety disorder reflect different facets of a single clinical entity. However, this paper will focus on the issue of smell, without expanding on this issue too much.

## EGORRHEA SYNDROME AND ADOLESCENT PARANOIA: *JIKOSHU* AS A DELUSION

In 1962,^20^ Akira Fujinawa and his colleagues coined the term “egorrhea” in their study of the *Jikoshu* experience, which encompasses a group of symptoms in which “something leaks out from oneself and becomes known to others or affects others.”^21^ To our knowledge, the term's earliest mention in the literature was in 1967,^22^ when Fujinawa proposed the term “egorrhoe” to signify that something in the ego leaks out, following the example of rhinorrea, liquorrhea, or logorrhea. However, given the term's Germanic spelling, “egorrhea” is more appropriate in English.

Patients with *Jikoshu* have delusional beliefs that their bodies emit unpleasant odors that cause others to dislike and avoid them. Over the years, symptoms in no small number of patients persist without developing into other psychiatric conditions.

These constant symptoms include (a) the delusion of soliloquy, in which the patient complains that they have spoken to themself unknowingly in public and that people know what they are thinking, (b) the delusion of talking in one's sleep, in which the patient says they have spoken secrets aloud in their sleep, and (c) a kind of thought broadcasting, in which the patient's thoughts leak out by themselves and are transmitted to others and become known. This series of symptoms has an experiential structure that can be summarized by the formula “something leaks out from oneself and is known to others or affects others.” This something might be a self‐odor, a soliloquy, sleep‐talk, or thought content. In mild cases it may be a blush, an ugly facial expression, or one's own gaze. Fujinawa considered these a series of clearly outlined symptoms that he called the egorrhea syndrome.

Egorrhea symptoms are found in a variety of disorders, but Fujinawa termed those found in schizophrenia “egorrhea schizophrenia,” the characteristics of which are summarized in Table 2.^20^ In presenting this concept, Fujinawa attempted to dichotomize schizophrenia into two series, one centered on “syndrome of influence” and the other on egorrhea syndrome. The term syndrome of influence is derived from *délire dꞌinfluence* in French psychiatry, which means “delusion characterized by the belief that outsiders exert an occult influence on the patient.”^23^ Thus, Fujinawa attempted to divide schizophrenia into two symptom clusters: one in which something inside the patient leaks out and harms others, and another in which something outside enters and harms the patient.

**Table 2:** Characteristics of egorrhea schizophrenia (Fujinawa).^20^, ^21^

1. Primarily thought broadcasting, thought echoing, delusions of sleep‐talking, and delusions of soliloquies, with transient *Jikoshu‐Kyofu* and *Jiko‐Shisen‐Kyofu*. 2. No syndrome of influence. 3. With abnormal cenesthesia. 4. Associated with guilt theme but no persecution theme. 5. Delusions are not systematic. 6. Symptoms persist for long periods of time but without a tendency toward personality disorganization. 7. Also seen in severe neurosis and borderline cases.

The symptoms that comprise egorrhea syndrome are delusional beliefs that something is leaking from oneself, therefore *Jikoshu* in this syndrome would also be considered a delusion. Although egorrhea syndrome can be seen in various mental disorders, Fujinawa seemed to be most concerned with schizophrenia. On the other hand, Murakami's group, contemporaries of Fujinawa, regarded similar symptoms as a delusional syndrome distinct from schizophrenia.

Murakami's group began their research around 1964 by distinguishing schizophrenia from *Jikoshu* experience, fear of looking at others, and dysmorphophobia, which their colleagues had diagnosed as schizophrenia or early‐stage schizophrenia at that time.^24^ Given that most cases with these symptoms occurred in adolescence and involved adolescent mentality, the condition was thought to have an affinity with paranoia, so it was named adolescent paranoia.^25^ Table 3 shows clinical features of adolescent paranoia according to Murakami.^24^ He states that adolescent paranoia has a wide range of adjacent pathologies, from neurotic to psychotic disorders. As a hypochondriasis, it exhibits a unique pathology specific to interpersonal situations, it is distinguished from phobia and obsessive‐compulsive disorder by its certainty, it has continuity with *Taijin‐Kyofu* in the dependence of symptoms on interpersonal situations but is distinguished from *Taijin‐Kyofu* by the presence of delusions of reference, the delusions of reference seen in this condition are limited to the patient's physical abnormalities and differ from delusions in schizophrenia, its monosymptomatic delusions are similar to those of delusional disorders but without systematic development, and the guilt expressed in this condition is not a feeling of guilt for one's actions, as in depression, but a feeling of being sorry because one's physical defects cause discomfort in others.^24^ Thus, Murakami concluded that adolescent paranoia is a clinical entity with a relatively clear symptom profile and stable structure, although it exhibits a “borderline nature” that is close, but not identical to, other diseases.

**Table 3:** Characteristics of adolescent paranoia (Murakami).^24^

1. Patients harbor a delusional belief that they cause others discomfort due to certain physical abnormalities of their own, such as their own body odor or the way they look at others. 2. As a result, they have delusions of reference (delusions of avoidance), such as being “disliked” or “avoided” by others along with accompanying feelings of guilt. 3. These symptoms are situation‐dependent and uttered or intensified only in the presence of others. 4. Patients generally are willing to be treated, but they seek their perceived abnormalities in physical illness and relentlessly demand corresponding treatment. 5. Most cases begin in adolescence, with symptoms monosymptomatic and without personality disorganization.

To summarize the studies presented in this section, the issue of *Jikoshu* and its proximate pathology's transition to schizophrenia was addressed by the egorrhea syndrome studies, and their close association with adolescent psychology was addressed by the adolescent paranoia studies.

## NOSOLOGICAL SINGULARITY OF *JIKOSHU*

In Tadao Miyamoto's 1976 article,^2^ he noted that contemporaneous studies in different countries showed a considerable nosological spread. Whereas the Japanese studies described above considered *Jikoshu* to be in proximity to *Taijin‐Kyofu*, in Germany *Jikoshu* was explained in relation to schizophrenia^26^, ^27^, ^28^ or cerebral organic diseases,^29^ and in France it was contrasted with neurasthenia or melancholy.^30^, ^31^

Fujinawa contrasted egorrhea syndrome with the syndrome of influence, although there are other references to similar directions of experience for olfactory symptoms. Miyamoto introduced the terms *égocentrique* (egocentric) and *exocentrique* (exocentric) from French psychiatry^30^, ^32^ as well as Tellenbach's terms *rezeptiv* (receptive) and *emanativ* (emanative).^33^ As we have seen, the “from‐self‐to‐surroundings” mode of experience or *égocentrique/rezeptiv* mode is prominent in the *Jikoshu* experience. On the other hand, in the “from‐surroundings‐to‐self” mode of experience or *exocentrique/emanativ* mode odor complaints, for example, are described as “strange smells wafting toward me from the surroundings,” with this direction usually observed in delusions of persecution in schizophrenia. This is not to say that there are no exocentric moments in *Jikoshu*. Many patients first experience exocentric experiences as people around them expressing their discomfort or avoiding them before perceiving their bodies emitting unpleasant odors. In other words, Fujinawa may have considered the pathogenesis of schizophrenia in two directions, whereas Miyamoto emphasized these two directions as inseparably linked in *Jikoshu* experience.

*Jikoshu* experience is situation‐dependent. The patient is unaffected when alone or with their family and affected only in the presence of classmates, neighbors, or even strangers. The patient with *Jikoshu* worries about anonymous people, but not other persons of particular significance who are present, as would be the case in the delusions of schizophrenia. Miyamoto believed that this explains why a patient with *Jikoshu* consistently harbors strong delusional convictions without exhibiting any specific delusional development.

Even so, he presented three types of *Jikoshu* experiences, with three distinct poles. The first is the hallucination type. Although some researchers^34^ attempted to divide *Jikoshu* experiences into olfactory delusions and olfactory hallucinations, all agree that *Jikoshu* is not a genuine hallucination but rather a unique experience that is closer to a delusion. Nonetheless, there are cases where the patient clearly states that they can smell their own body. In these cases, egocentric orientation prevails and the patient complains that they can smell themself even when alone. These are transient symptoms with a good prognosis.

The second is the reference type, which is an exemplar of a *Jikoshu* experience. The mechanism of relating others' reactions to one's own odor is recognized in the establishment of symptoms, with the experience naturally preceded by an exocentric direction. The tendency to self‐reference persists but rarely develops into a genuine delusion of reference and lacks the element of olfactory hallucinations. The individual provides their own explanations or interpretations of why body odors occur. These explanations do not deviate greatly from common sense, although in some cases they become bizarre and develop into the next type.

The third is the cenesthetic type. Here, abnormalities of cenesthesia are wide ranging, from mild abnormalities of bodily sensation to cenesthopathy, or from bodily hallucinations to bodily delusions, with all characterized by the addition of bizarre nuances. The direction of the experience is not clear, with a mixture of egocentric and exocentric directions. Compared with the first two types, patients are often older (30s or older). They look inside their own bodies and attempt to find various explanations for their body odor. Despite their bizarre bodily delusions, their communication is good and they do not speak or act abnormally apart from the subject of *Jikoshu*. However, they are convinced that their bodies are abnormal, so they visit various departments outside of psychiatry. They are relentless in their efforts to realize their intentions. For example, a patient who claims that her body odor is caused by odor leaking from a hole in her uterus might seek a gynecologist to remove her uterus.

From this perspective, Miyamoto stated that the *Jikoshu* experience “remains an entity of psychopathology that is itself extremely significant and has the potential to develop into various pathological phenomena, while maintaining an equidistant relationship with neurosis, schizophrenia, and manic‐depressive illness, respectively.”^24^ Thus, he suggested calling the condition *Jikoshu‐Sho* because adding words such as “fear,” “hallucination,” or “delusion” to a disease that presents with *Jikoshu* could undermine the totality of this experience.

## RISE OF OLFACTORY REFERENCE DISORDER

Like *Taijin‐Kyofu*, *Jikoshu‐Sho* has been considered a culture‐bound syndrome unique to Japan or East Asia.^35^, ^36^ However, Miyamoto noted that descriptions of similar symptoms exist in the German‐ and French‐language literature.^2^ In the English‐language literature, the earliest reference to this syndrome dates back to 1891.^37^ However, it seems unlikely that there were no such cases in the West, but without the proper terminology, such cases would be difficult to report. Such a term was first presented in the English‐language literature in Pryse‐Phillips' 1971 article on olfactory reference syndrome (ORS).^38^ However, that article initially received little attention: a PubMed search for articles with ORS in the title found only five before 1983, although the number of articles increased after 1997. Suzuki et al.^35^ stated that *Jikoshu‐Kyofu* and ORS share a common clinical entity, with ORS considered a counterpart of *Jikoshu‐Kyofu* in the English‐language literature. ORS has been defined as a psychiatric condition characterized by persistent preoccupation with body odor accompanied by shame, embarrassment, significant distress, avoidance behavior, and social isolation.^39^

The revival of ORS is due to the adoption of obsessive‐compulsive spectrum disorder (OCSD),^40^ proposed by Hollander et al., in the *Diagnostic and Statistical Manual of Mental Disorders*, 5th edition (DSM‐5). In establishing “obsessive‐compulsive and related disorders” in the DSM‐5,^11^ body dysmorphic disorder, hoarding disorder, trichotillomania (hair‐pulling disorder), and excoriation were listed as “related disorders” in addition to OCD, with reference to the OCSD concept of preoccupation and repetitive behaviors as the basic pathology. Initially considered for inclusion in the OCD category in the DSM‐5,^37^ ORS was dropped due to lack of evidence establishing it as a separate disorder. ORS was listed with only the remark on *Jikoshu‐Kyofu* in the “other identified obsessive‐compulsive and related disorder” in the DSM‐5. Finally, the ICD‐11 (*International Classification of Diseases 11th Revision*)^41^ adopted ORS as an olfactory reference disorder in consideration of its clinical usefulness.

However, from our perspective, there are problems with this. In OCSD, the concept of obsession/compulsion has already been transformed from the concept of *Zwang* (obsession/compulsion) developed in German psychiatry as Schneider's description below clearly shows. According to Schneider, compulsion can only be defined with regard to a central concept: “With these reservations, we would say that compulsion may be said to occur when an individual is haunted by conscious contents, although at the same time he judges them as senseless or at any rate senselessly insistent.”^42^ Of course, this “judgment of senselessness” is an important issue in Japanese psychiatry, as well in French psychiatry as stated in the entry for “*obsessions et névrose obsessionnelle*” in Porot's French‐language psychiatric dictionary: *le sujet ne peut haser bien quꞌil les tienne pour absurdes* (the patient cannot chase them away even though he holds them to be absurd).^43^ The DSM‐IV OCD diagnostic criteria included the following sentence, which was removed in the DSM‐5: “At some point during the course of the disorder, the person has recognized that the obsessions or compulsions are excessive or unreasonable.”^10^ Starcevica and Janca correctly pointed out that the “essence of compulsive behaviour in OCD was suggested to specifically reside not in its repetitiveness, but in its function to alleviate obsession‐induced anxiety and distress; on the contrary, the function of compulsion‐like behaviours in most OCSDs is to provide pleasure or gratify an urge, an impulse, or a need.”^44^ Thus, obsession/compulsion in OCSD and OCD in the DSM‐5 represent something different from the classical concept.

Nevertheless, this paper is concerned with *Jikoshu‐Sho*, which, as seen already, does not fit into the classical obsession and often naturally develops into delusions of reference or cenesthopathy without “judgment of senselessness.” On the other hand, despite the preoccupation with body odor, *Jikoshu‐Sho* usually shows no significant repetitive behavior, calling into question its inclusion in the category of OCSD. The perspective that ORS/*Jikoshu‐Sho* is a condition that occurs in an interpersonal setting, as is the case with body dysmorphic disorder, is scarcely discussed in OCSD. ORS/*Jikoshu‐Sho* is inevitably accompanied by perceived annoyance to and avoidance by surrounding people due to one's own body odor, and cannot be fully encompassed by OCSD. In this respect, the association with social anxiety disorder^45^ is an important issue, and social anxiety disorder should be extended to include conditions with delusions of reference and cenesthopathy, as in the case of *Taijin‐Kyofu*. Although the incorporation of ORS into international diagnostic criteria would be welcomed, a reorganization of clinical entities would be required to properly place *Jikoshu* within the diagnostic criteria. The author believes that the diagnostic criteria for OCSD (and OCD in the DSM‐5) would be overinclusive or inaccurate and those for social anxiety disorder would be underinclusive.

## AUTHOR CONTRIBUTION

Toshiyuki Kobayashi is the only author of the review.

## CONFLICT OF INTEREST STATEMENT

The author declares no conflict of interest.

## ETHICS APPROVAL STATEMENT

Since this study was a review, ethical approval was not necessary.

## PATIENT CONSENT STATEMENT

N/A

## CLINICAL TRIAL REGISTRATION

N/A

## Data Availability

N/A
